# Reinforcement of bacterial cellulose aerogels with biocompatible polymers

**DOI:** 10.1016/j.carbpol.2014.04.029

**Published:** 2014-10-13

**Authors:** N. Pircher, S. Veigel, N. Aigner, J.M. Nedelec, T. Rosenau, F. Liebner

**Affiliations:** a University of Natural Resources and Life Sciences Vienna, Division of Chemistry of Renewables, Konrad-Lorenz-Straße 24, A-3430 Tulln, Vienna, Austria; b University of Natural Resources and Life Sciences Vienna, Department of Wood Science, Konrad-Lorenz-Straße 24, A-3430 Tulln, Vienna, Austria; c Clermont Université, ENSCCF, Institute of Chemistry of Clermont-Ferrand, BP 10448, 63000, Clermont-Ferrand, France; d CNRS, UMR 6296, ICCF, 24 av. des Landais, 63171 Aubière, France

**Keywords:** Bacterial cellulose, Cellulosic aerogels, Cellulose composite materials, Interpenetrating polymer networks, Reinforcement, Supercritical carbon dioxide

## Abstract

Bacterial cellulose (BC) aerogels, which are fragile, ultra-lightweight, open-porous and transversally isotropic materials, have been reinforced with the biocompatible polymers polylactic acid (PLA), polycaprolactone (PCL), cellulose acetate (CA), and poly(methyl methacrylate) (PMMA), respectively, at varying BC/polymer ratios. Supercritical carbon dioxide anti-solvent precipitation and simultaneous extraction of the anti-solvent using scCO_2_ have been used as core techniques for incorporating the secondary polymer into the BC matrix and to convert the formed composite organogels into aerogels.

Uniaxial compression tests revealed a considerable enhancement of the mechanical properties as compared to BC aerogels. Nitrogen sorption experiments at 77 K and scanning electron micrographs confirmed the preservation (or even enhancement) of the surface-area-to-volume ratio for most of the samples.

The formation of an open-porous, interpenetrating network of the second polymer has been demonstrated by treatment of BC/PMMA hybrid aerogels with EMIM acetate, which exclusively extracted cellulose, leaving behind self-supporting organogels.

## Highlights

• Reinforcement of cellulose aerogels with biopolymers PLA, PCL, CA and PMMA.
• Interpenetrating and open porous networks of cellulose and biocompatible polymers.
• scCO_2_ anti-solvent precipitation and extraction used as core techniques.
• Cellulose aerogels used as template for the preparation of porous PMMA scaffolds.

## 1 Introduction

Bacterial cellulose (BC) is an extracellular natural byproduct of the metabolism of various bacteria (Deinema & Zevenhuizen, 1971), with *Acetobacter* spp. strains being most commonly used. BC is produced by the respective bacteria strains in response to specific environmental conditions. *Acetobacter xylinum*, for example, produces cellulose pellicles that keep the bacterium floating on the surface to maintain sufficient oxygen supply. Other bacteria, such as the plant pathogen *Agrobacterium tumefaciens*, use cellulose for better attachment to plants, similar to the symbiotic *Rhizobium* spp.

Bacterial cellulose, grown under controlled conditions on appropriate carbon and nitrogen sources, forms highly porous network structures, whose voids are filled with the culture medium. The macroscopic appearance (pellicles, sheets, tubes, etc.) varies depending on the technological approach (static vs. agitated, batch vs. continuous cultivation, rotary vs. disk fermenters, e.g.). After removing the culture medium and thorough washing, a tasteless, colorless, and odorless translucent and chewy gel is obtained which, to date, is mainly commercialized as a dietary auxiliary. However, applications in skin care (Nanomasque^®^; Amnuaikit, Chusuit, Raknam, & Boonme, 2011) and topological wound healing (Suprasorb^®^X, Bioprocess^®^, XCell^®^, and Biofill^®^; Petersen & Gatenholm, 2011), which both take advantage of the high purity of BC, its positive effect on skin tissue regeneration (Sutherland, 1998) and its great water-retaining and moisturizing capabilities, are currently advancing strongly. Beyond that, good biocompatibility and low immunogenic potential (Helenius et al., 2006; Klemm, Schumann, Udhardt, & Marsch, 2001) render BC a promising material for various biomedical applications. This comprises their use as artificial blood vessels (Klemm et al., 2001), semi-permanent artificial skin (Petersen & Gatenholm, 2011), as well as matrices for slow-release applications (Haimer et al., 2010), nerve surgery (Klemm et al., 2001), engineering of bone tissue (Zaborowska et al., 2010) or artificial knee menisci (Bodin, Concaro, Brittberg, & Gatenholm, 2007).

Quantitative replacement of water by an organic solvent and subsequent extraction of the organic solvent from the porous BC matrix with supercritical carbon dioxide (scCO_2_) has been demonstrated to be the most successful approach for converting BC hydrogels into the respective aerogels. The solvent must be miscible with both water and scCO_2_, as it is the case for, e.g., ethanol or acetone. This drying procedure preserves the fragile cellulose network structure and the hierarchical system of micro-, meso, and macropores (Liebner et al., 2010; Maeda, Nakajima, Hagiwara, Sawaguchi, & Yano, 2006b). Bacterial cellulose aerogels feature an outstandingly low bulk density in dry state (≥10 mg cm^−3^), have low heat transmission and thermal expansion coefficients, are fully re-hydratable and share all of the above properties relevant for biomedical applications. Therefore, BC aerogels expand the scope of BC applications considerably, be it in terms of sensing (e.g. by quantum dots), thermal or acoustic insulation, specific sorption (from gases or liquids), catalysis, or slow release of active compounds.

However, despite the high tensile modulus and strength of individual BC ribbons (>10 GPa and >17 MPa, respectively, Svensson et al., 2005), the resistance of BC aerogels and their hydrated precursors towards compressive mechanical stress is not sufficiently high for many applications that involve mechanic wear. Numerous reinforcing strategies have been therefore investigated, including preparation of all-cellulose composites, controlling fibril properties by adding special additives to the nutrient medium, incorporation of strength-imparting polymers during BC growth, chemical surface modification, or cross-linking (Seifert, Hesse, Kabrelian, & Klemm, 2004; Yano, Maeda, Nakajima, Hagiwara, & Sawaguchi, 2008).

Three-dimensional networks of a secondary polymer interpenetrating and reinforcing that of bacterial cellulose can be prepared by soaking BC with a solution of the respective monomer and covalent grafting onto BC (e.g. BC-*g*-PMMA, BC-*g*-PBA, BC-*g*-PMMA-*co*-PBA; Lacerda, Barros-Timmons, Freire, Silvestre, & Neto, 2013). Further techniques are in situ generation of the interpenetrating network by loading and subsequent chain-growth polymerization of a suitable monomer such as methacrylic acid (Hobzova, Duskova-Smrckova, Michalek, Karpushkin, & Gatenholm, 2012) or precipitation of the reinforcing polymer from a compatible solvent, filling the voids of the cellulosic network, as it has been described in our previous work for BC/cellulose acetate composites (Liebner, Aigner, Schimper, Potthast, & Rosenau, 2012). BC hybrid materials containing an inorganic polymer have been obtained by loading of silica sol into (Yano et al., 2008) or polymerization of silicate precursors within the BC structure (Maeda, Nakajima, Hagiwara, Sawaguchi, & Yano, 2006a). Another process that affords organic/inorganic hybrid materials is biomineralization of appropriately functionalized cellulosic scaffolds, as it takes place in (simulated) body fluids (Zimmermann, LeBlanc, Sheets, Fox, & Gatenholm, 2011).

The majority of previous studies used the above approaches either to reinforce thin BC films directly or to obtain mechanically resistant BC sheets from modified bulk BC organogels after compaction. However, to make use of the intriguing native morphology of three-dimensional BC aquogels, the reinforcing approaches should aim at a far-reaching preservation of the inherent BC cellulose network architecture.

The current study investigates the reinforcement of BC aerogels with interpenetrating, biocompatible and partially biodegradable polymers, such as polylactic acid (PLA), polycaprolactone (PCL), cellulose acetate (CA) and poly(methyl methacrylate) (PMMA). The three-dimensional network of the entangled BC fibers has been studied as a template for the preparation of porous PLA-, PCL-, CA-, and PMMA scaffolds of BC-like morphology under preservation or enhancement of the surface-to-volume ratio. Supercritical carbon dioxide anti-solvent precipitation and extraction, respectively, have been used as core techniques for depositing the secondary polymer within the BC matrix and to convert the formed composite organogels into aerogels.

## 2 Materials and methods

PLA was obtained from NatureWorks LLC (PLA Polymer 4042D; *M*_w_ 209.0 kg mol^−1^, 6.1% D-isomer). PCL (*M*_w_ 48.0–90.0 kg mol^−1^, *M*_n_ ∼45.0 kg mol^−1^), CA (*M*_n_ ∼30.0 kg mol^−1^, 39.8 wt% acetyl) and PMMA (*M*_w_ ∼350.0 kg mol^−1^) were purchased from Sigma-Aldrich (Vienna, Austria). Absolute ethanol was obtained from Fisher Scientific (Vienna, Austria). Tetrahydrofuran (HiPerSolv CHROMANORM for HPLC) and acetone (AnalaR NORMAPUR) were obtained from VWR (Vienna, Austria).

### 2.1 Preparation of bacterial cellulose

Bacterial cellulose was kindly provided by the Research Centre for Medical Technology and Biotechnology (FZMB) Bad Langensalza, Germany. The material was produced by a static cultivation of *Gluconacetobacter xylinum* AX5 wild type strain on Hestrin-Schramm growth medium for 30 days at 30 °C.

The obtained BC layer was cut into 120 mm × 20 mm × 20 mm cuboids, heated three times for 20 min in 0.1 M aqueous NaOH at 90 °C, and finally rinsed with deionized water for 24 h. Afterwards the BC was subjected to a solvent exchange, replacing water by 96% ethanol.

### 2.2 Preparation of BC-based composite aerogels

Prior to modification, the BC was cut into smaller cuboids featuring edge lengths of about 10 mm. Considering the transverse isotropy of BC aerogels (Liebner, Aigner et al., 2012) and with respect to the evaluation of the mechanical properties of the composites, the specimens were marked along the direction of the 120 mm edges of the parent BC samples. These edges correspond to one of the horizontal (plane) directions of the harvested BC and are perpendicular to their (weaker) growth direction.

The respective BC specimens were transferred first to tetrahydrofuran (in the case of PCL and PLA) or acetone (in the case of CA and PMMA), corresponding to the type of solvent used for dissolution of the reinforcing polymer, and subsequently into the loading baths which contained solutions of the respective reinforcing polymer at overall concentrations of 10, 20, 40, 80 and 120 mg mL^−1^ (sample labeling refers to these concentrations, e.g.: PLA10). All solvent exchange and loading steps were carried out in total volumes corresponding to the ten-fold volume of the respective BC sample. After a residence time of at least 24 h at room temperature (PCL, CA, PMMA) and 50 °C (PLA), respectively, the samples were removed from the loading bath. Precipitation of the second polymer within the BC pore network was carried out with either ethanol (in the case of PLA and PCL) or scCO_2_ (for CA and PMMA). Conversion of composite organogels to the respective aerogels was in either case accomplished by scCO_2_ drying: The organogels were placed into a 300 mL autoclave equipped with a separator for carbon dioxide recycling (Separex, France). Drying was performed under constant flow of scCO_2_ (40 g min^−1^) at 10 MPa and 40 °C for two to three hours. The system was then slowly and isothermally depressurized at a rate of <0.1 MPa min^−1^.

### 2.3 Characterization of BC aerogels and BC composite aerogels

Shrinkage of the organogels during loading/precipitation and subsequent drying was determined by measuring the dimensions and calculating the volume of the cuboids before loading with the respective polymer solution and after scCO_2_ drying. To calculate densities, the weight of the aerogels was determined gravimetrically after drying.

Scanning electron microscopy (SEM) of gold sputtered samples (Leica EM SCD005 sputter coater, layer thickness 6 nm) was performed on a Tecnai Inspect S50 instrument under high vacuum at an acceleration voltage of 5.00 kV.

Polarized light microscopy was performed on a Leica DM4000 M microscope. Images were recorded with a digital camera (Leica Microsystems Wetzlar GmbH, Germany).

Thermoporosimetry was conducted on a Mettler-Toledo DSC30 instrument equipped with a liquid nitrogen module calibrated (both for temperature and enthalpy) with metallic standards (In, Pb, Zn) using *o*-xylene as interstitial liquid. About 10 or 20 mg of the studied material was placed into a DSC pan which was then sealed and subjected to repeated freezing/thawing cycles in comparison to an empty DSC pan. A detailed description of the applied temperature program can be found elsewhere (Bruns, Lallemand, Quinson, & Eyraud, 1977; Nedelec, Grolier, & Baba, 2006).

Mechanical response profiles towards compressive stress orthogonally to the (weaker) growth direction of BC were recorded on a Zwick-Roell Materials Testing Machine Z020. The required strain to achieve a deformation speed of 2.4 mm min^−1^ was measured in a 500 N load cell. Yield strength (*R*_P0.2_) was defined as the stress at 0.2% plastic deformation.

Nitrogen adsorption/desorption isotherms at 77 K have been obtained on a Micromeritics ASAP 2020 analyzer. All samples were degassed in vacuum prior to analysis. Specific surface areas were calculated using the Brunauer, Emmett and Teller (BET) equation.

## 3 Results and discussion

### 3.1 Shrinkage and bulk density

Static cultivation of *Acetobacter xylinum* AX 5 wild type strain on Hestrin-Schramm medium affords bacterial cellulose aquogels that can be converted into the respective aerogels at very low shrinkage (1–5%) by scCO_2_ treatment (supercritical point of CO_2_: 31.2 °C, 7.38 MPa), if the interstitial water is quantitatively replaced by an appropriate CO_2_-miscible organic solvent prior to the drying step (40 °C, 10 MPa; Liebner et al., 2010; Maeda, 2006). Ethanol as a medium-polar solvent that is miscible with both H_2_O and CO_2_ is frequently used in scCO_2_ drying of aerogels. The morphology of polysaccharide-based gels, such as of BC aquogels, which consist of entangled ribbon-type cellulose microfibrils and interstitial water, can be largely preserved during this solvent exchange (ethanol) and the subsequent scCO_2_ drying. This is reflected by the low apparent density of the obtained aerogels (7.8 ± 0.5 mg cm^−3^; *n* = 5) which is in good agreement with values reported elsewhere (8.3 ± 0.7 mg cm^−3^; Liebner et al., 2010).

However, loading of BC gels with the reinforcing polymers poly(lactic acid) (PLA), polycaprolactone (PCL), cellulose acetate (CA), and poly(methyl methacrylate) (PMMA) required solvents other than ethanol due to solubility issues. While acetone was the solvent for CA and PMMA, tetrahydrofurane (THF) was used in the case of PLA and PCL. BC reference samples, which had been manufactured using the respective solvents acetone and THF instead of ethanol and which did not contain the reinforcing polymer, revealed that the type of organic solvent has a weak impact on the overall shrinkage of the gels during processing and hence on the apparent density and response of the obtained aerogels towards compressive stress. Compared to BC aerogels prepared from the respective alcogels (7.8 ± 0.5 mg cm^−3^), replacement of the interstitial ethanol by acetone prior to scCO_2_ drying afforded somewhat higher densities of 9.4 ± 0.6 mg cm^−3^ (reference samples CA0 and PMMA0; *n* = 4), similar to data reported elsewhere; Liebner, Aigner et al., 2012). The densities of aerogels obtained by sequential solvent exchange from ethanol to THF and back to ethanol prior to scCO_2_ drying (reference samples PLA0 and PCL0) were found to be in the same range (9.6 ± 0.8 mg cm^−3^; *n* = 4).

The impact of the type of solvent on the apparent density of the aerogels is most likely due to the different strengths of interactions that occur between the respective solvents and the surface of the cellulose microfibrils and are strongly influenced by the abundance of OH groups. According to the Hansen model of solvent–polymer interactions, the cohesive energy density (expressed as Hildebrand solubility parameter) can be calculated as the sum of a dispersion force component, a polar component and a hydrogen bonding component. Replacement of ethanol (*δ*_SI_ = 26.5 MPa^1/2^) by acetone or THF decreases the total Hildebrand parameter to *δ*_SI_ = 20.0 MPa^1/2^ and *δ*_SI_ = 19.4 MPa^1/2^, respectively. The hydrogen bonding component, which is, due to the high abundance of OH groups, supposed to be of particular importance for solvent–polymer interactions, is even more affected and decreases from *δ*_H_ = 19.4 MPa^1/2^ (ethanol) to 8.0 MPa^1/2^ (THF) and 7.0 MPa^1/2^ (acetone), respectively. A similar effect is assumed to occur in the initial phase of scCO_2_ drying, when CO_2_ and solvent form a rather non-polar, expanded liquid phase inside the pores of the gels to be dried. This is evident from the extensive shrinking that has been reported for the preparation of aerogels from a variety of biopolymers (starch, alginate, cellulose).

**Fig. 1:**
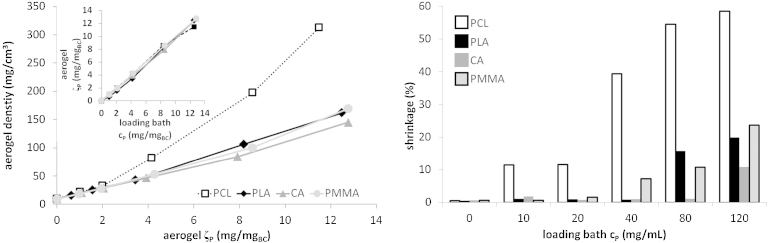
Bulk density of reinforced BC aerogels vs. mass ratio of the secondary polymer (*ζ*_p_) in the aerogel (A; inset: *ζ*_p_ in the aerogel vs. concentration of the secondary polymer in the loading bath (*c*_p_)). Overall shrinkage of gels during loading, solvent exchange and scCO_2_ extraction vs. loading bath concentration (B).

Corresponding to the rather marginal, solvent-dependent differences in shrinkage, the spatial dimensions of BC specimen were largely preserved throughout loading and precipitation of PLA, CA and PMMA, and during scCO_2_ drying of the BC/PLA, BC/CA and BC/PMMA hybrid organogels, in particular for those variants with low polymer concentration in the loading baths. Stronger shrinkage was observed only at concentration levels of 80 and 120 mg mL^−1^, corresponding to a polymer-to-BC mass ratio of ≥7.9, with the highest value observed for PMMA120 (23.7%, Fig. 1).

Reinforcement of cellulose aerogels with polycaprolactone (PCL) through ethanol anti-solvent precipitation from THF and subsequent scCO_2_ drying, turned out to be a less feasible approach as substantial collapsing of the specimen occurred during scCO_2_ drying. This effect, which was observed for all PCL/BC hybrid organogels exceeding a PCL/BC mass ratio of 2.0 (*cf*. Fig. 1), is triggered by the comparatively strong expansion of PCL under scCO_2_ conditions, caused by the low glass transition temperature (*T*_G_) of PCL (−60 °C) and the good solubility of CO_2_ in PCL, which exists in a rubbery state under the conditions employed. While fast depressurization of CO_2_-expanded neat PCL affords stable foams (Xu et al., 2004), slow depressurization rates of less than 0.1 MPa min^−1^ – as typically used to preserve the fragile cellulose network structure of respective organogels (Liebner et al., 2010) – causes the expanded PCL rubber to collapse. As a result, compact BC hybrid materials with densities about twice as high as observed for all other hybrid aerogels were obtained with cellulose ribbons stuck together by PCL. Interestingly, collapsing of the network structure was much more pronounced along the growth direction of BC, indirectly confirming their anisotropic morphology and response towards mechanical stress (see below). A similar shrinkage effect was not observed for PLA (*T*_G_ = 55–60 °C), PMMA (*T*_G_ = 90–105 °C; Buck, Diem, Schreyer, & Szigeti, 1975; Dixit et al., 2009) and CA (*T*_G_ = 140–190 °C; Vallejos, Peresin, & Rojas, 2012) whose glass transition temperatures are distinctly above the employed operation temperature of the scCO_2_ unit (40 °C).

With the exception of BC/PCL hybrid aerogels, the bulk densities of the obtained sets of reinforced BC aerogels ranged from 16 to 170 mg cm^−3^, depending on the respective concentration of the loading bath as well as the extent of shrinkage (Fig. 1). The amount of secondary polymer contained in a certain volume of the loading bath was also found in the obtained aerogel (Fig. 1A, inset), indicating the absence of specific interactions between BC and the loaded substances which could have caused an enrichment effect. In particular at higher loadings, BC composites with CA exhibited lower shrinkage rates and densities compared to the other organogels.

### 3.2 Morphology of BC hybrid aerogels

**Fig. 2:**
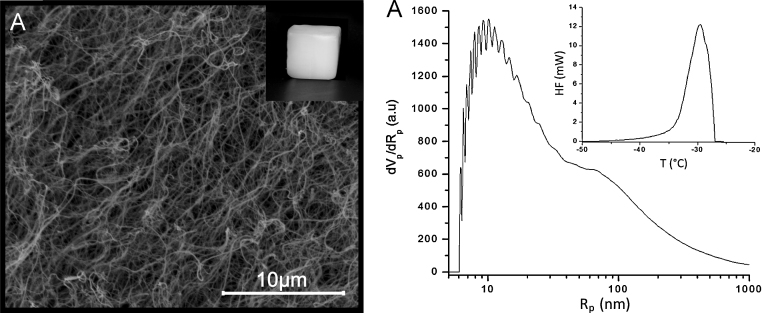
SEM picture of an unmodified BC aerogel at 10.000× magnification (A) and its void size distribution as analyzed by thermoporosimetry using *o*-xylene as confined solvent (B; inset: Thermogram of a deep-frozen BC aerogel soaked with *o*-xylene).

The ‘biological spinnerets’ lined up in BC producing bacteria release cellulose as an extracellular substance in the form of elementary fibrils which aggregate to ribbons. As the cellulose synthesizing sites are duplicated during cell division (Brown, Willison, & Richardson, 1976), mother and daughter cell are connected to one and the same cellulose ribbon which causes formation of a highly interconnected and entangled three-dimensional network of cellulose (Fig. 2A). Nitrogen sorption experiments at 77 K, the results of thermoporosimetry measurements (Fig. 2B) and SEM micrographs reveal a very broad pore size distribution with void diameters ranging from the single digit nano- to micrometer range.

**Fig. 3:**
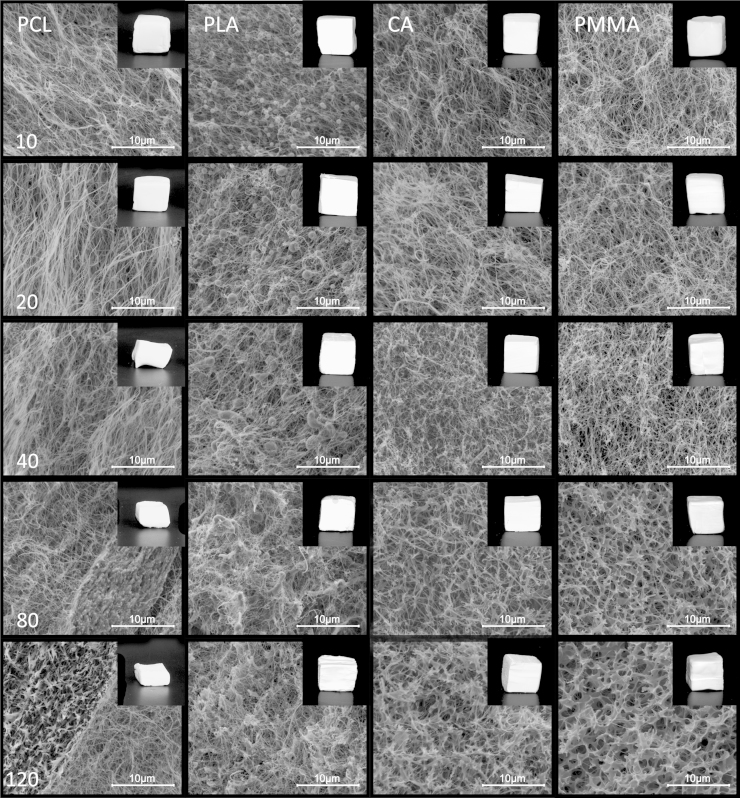
Scanning electron images of reinforced BC aerogels at 10.000× magnification. Numbers on the left side are referring to the concentration of the loading bath in mg mL^−1^.

The morphology of the aerogels changes gradually with increasing content of loaded polymer once a second component is introduced into this network (Fig. 3). The above-described agglutinative effect of PCL can already be seen at the lowest concentration level. At higher loadings the formation of distinctly separated regions, strongly deviating in their morphology are clearly visible in the respective SEM images. While some areas appear very similar to the original BC network, in others the reinforced fibers are agglomerated to clusters forming multi-layered structures, penetrating the composite perpendicular to the growth direction. These structures are associated with the collapse of the cuboids at higher loadings.

PLA is precipitated in form of small, individual spheres with particle sizes of about 0.5–2.0 μm (PLA10 and PLA20) or ellipsoids (PLA40) at lower PLA concentrations in the loading bath, corresponding to PLA/BC ratios of ≤3.5. At higher ratios (PLA80 and 120; PLA/BC: 8.2 and 12.5) a second, interpenetrating porous structure is formed within the BC aerogel. The establishment of this secondary network is also apparent from the response of these samples towards compressive stress, as both *E* modulus and yield strength increase significantly (Fig. 6, Table 1).

In contrast to the polyesters PLA and PCL, cellulose acetate and poly(methyl methacrylate) are evenly precipitated in close proximity to the surface of the BC fibrils. Up to a polymer/BC ratio of about 4 (concentration level ≤40 mg mL^−1^) the BC network acts as a template that governs the morphology of the secondary polymer network and supports the formation of highly open-porous composite materials with pore characteristics similar to those of pure BC aerogels. At polymer/BC ratios ≥5 the morphology of the obtained aerogels is increasingly dominated by the properties of the pervading polymer and resembles the open porous microstructure of pure CA or PMMA films obtained by various scCO_2_ processes (Reverchon & Cardea, 2004; Reverchon, Cardea, & Rappo, 2006; Soares da Silva, Viveiros, Coelho, Aguiar-Ricardo, & Casimiro, 2012).

**Fig. 4:**
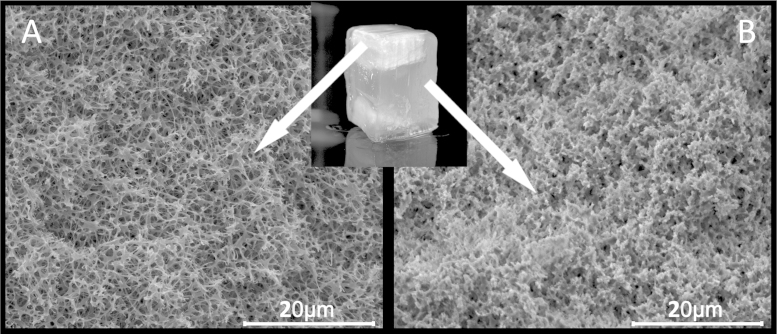
BC/PMMA80 organogel during extraction of BC with an ionic liquid, containing regions of varying amounts of residual BC (opaque). SEM pictures: morphology of a BC/PMMA80 aerogel (A) and of an aerogel as obtained from (A) after extraction of BC by EMIM acetate.

The formation of an open-porous, interpenetrating network of the second polymer was confirmed by treating selected BC/PMMA hybrid aerogels with the cellulose solvent 1-ethyl-3-methyl-imidazolium acetate (EMIM acetate). Even at a high PMMA/BC ratio of about 8, representing one of the least favorable case with regard to easiness of cellulose dissolution cellulose was extracted by the ionic liquid at 50 °C, leaving behind organogels which were largely transparent prior to drying and whose morphologies corresponded to those of the respective composites (Fig. 4). ATR-IR analysis of the extracted substance confirmed the extraction of pure cellulose (more than 90% of the amount of cellulose originally present in the composite aerogel) during this process (Figure S1, supplementary data).

### 3.3 Response towards compressive stress

**Fig. 5:**
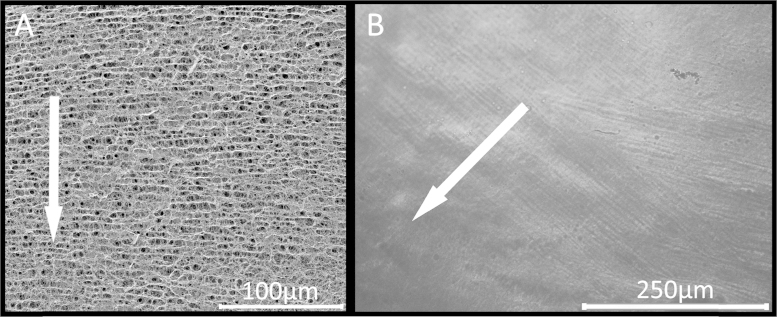
SEM picture of freeze-dried BC (A) and cross-polarization micrograph of a frozen BC sample (B). The white arrows indicate the direction of BC growth.

The anisotropic response of bacterial cellulose towards mechanical stress is an issue that must be considered in both characterization and utilization of BC-based materials. Static cultivation of BC-producing bacteria for 3–4 weeks typically affords a bacterial cellulose fleece of a few centimeters in thickness. However, as all BC producing bacteria strains are aerobic microorganisms, the release of cellulosic elementary fibrils and their aggregation to ribbons occurs only in close proximity to the phase boundary between culture medium and air. It is known that the density of BC films thus varies in the range of micrometers from top to bottom (Bodin, Bäckdahl et al., 2007). Over time, gravity pulls the thickening cellulose fleece deeper into the culture medium which is assumed to happen in micro steps. As a result, a transversely isotropic bacterial cellulose network is produced, which features a higher density and degree of entanglements parallel to the liquid/air phase boundary than in growth direction. Even though the less pronounced entanglement and density of BC ribbons in growth direction is not directly evident from respective SEM or ESEM pictures, it can be indirectly visualized by scanning electron microscopy of freeze-dried material (Fig. 5A) or polarization microscopy of frozen BC samples (Fig. 5B). During freezing the expanding water pushes the cellulose network along its weaker direction apart, forming alternating layers of compacted and less compacted cellulose ribbons. Depending on the freezing conditions the distance between those compacted cellulose layers is in the lower micrometer range (3–10 μm) which is in accordance with the literature (Bodin, Bäckdahl et al., 2007).

The significantly lower stiffness and strength of BC sheets in the direction of growth is also evident from the anisotropic response of BC aerogels towards compressive stress. The latter were obtained by scCO_2_ drying (40 °C, 10 MPa, 3 h) of respective BC samples after replacing the interstitial water by ethanol. While a Young's modulus of *E* = 0.057 ± 0.007 MPa and yield strength of *R*_P,0.2_ = 4.65 ± 0.48 kPa was observed along the growth direction, the respective values for the other two spatial directions were significantly higher (A: *E* = 0.149 ± 0.023 MPa, *R*_P,0.2_ = 7.05 ± 0.55 kPa; B: *E* = 0.140 ± 0.036 MPa, *R*_P,0.2_ = 7.84 ± 1.06 kPa). Because of this anisotropy, mechanical testing of all prepared BC composite aerogels was performed by applying the respective compressive stress along one of its stronger directions, which can be unambiguously identified by the long edges of the supplied BC samples (ca. 120 × 20 × 20 mm), which do not represent the growth direction.

**Fig. 6:**
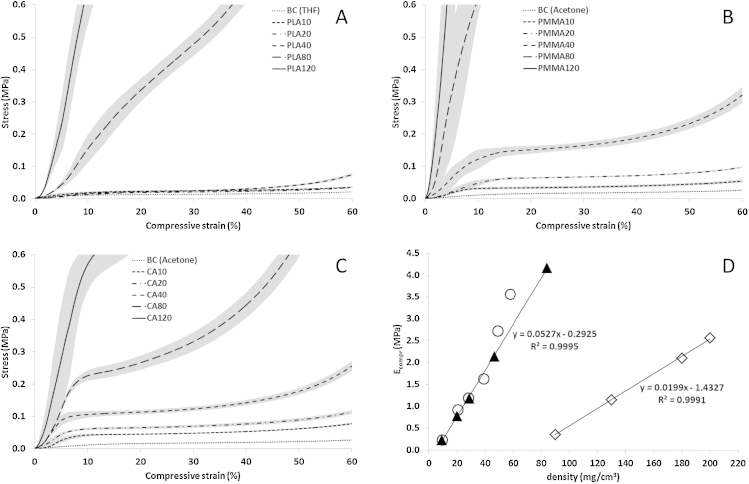
Mechanical response profiles towards compressive stress for BC aerogels reinforced with PLA (A), PMMA (B) and CA (C). Grey areas indicate standard deviations. (D) Correlation between Young's modulus and bulk density of BC/CA composites (triangles: CA0-80; circles: values from Liebner, Aigner, Schimper, Potthast, & Rosenau, 2012) and silica aerogels (diamonds: values from Alaoui, Woignier, Scherer, & Phalippou, 2008).

The mechanical response profiles of BC/PLA, BC/CA and BC/PMMA composite aerogels towards compressive stress are largely similar to those of aerogels prepared from pure BC, at least up to a polymer/BC ratio of about 4 (Fig. 6A–C; for full response curves of those BC aerogels that were reinforced with the highest amounts of PLA (A), PMMA (B) and CA (C) see Figure S2). They are characterized by an adjustment phase (≤3–5% strain), in which sample irregularities are evened out, followed by a comparatively narrow range of linear elastic deformation (<10%). The most eye-catching feature however is the pronounced plateau region (15–40%), caused by plastic deformation through cell collapsing and eventually followed by an exponential increase of stress over strain due to material densification. Similar to aerogels from regenerated cellulose (Liebner, Dunareanu et al., 2012) – and in contrast to brittle foams and silica aerogels – all composite aerogels deformed in a ductile way on the microscale.

While the stiffness of the BC/CA and BC/PMMA aerogels (Fig. 6B and C) significantly increased with each ascending concentration level, the same effect was observed for BC/PLA only for the two highest concentrations (Fig. 6A). The most regular reinforcing effect was observed for the BC/CA composites, where the *E* modulus increased linearly up to a CA/BC ratio of 8 (CA80; Fig. 6D) which is in good agreement with one of our previous studies (Liebner, Aigner et al., 2012).

The quotient of Young's modulus and bulk density (specific modulus *E*_ρ_) is a convenient parameter to compare the stiffness of materials of varying density. For pure BC aerogels it was calculated to be 19 (ethanol), 21 (THF) and 25 MPa cm^3^ g^−1^ (acetone; Table 1) which is remarkably high compared to other porous materials. For a polyurethane foam of a density of 90 mg cm^−3^, which is comparable to composites with a polymer/BC ratio of 8, a specific modulus of 7.8 MPa cm^3^ g^−1^ has been previously reported (Patel, Shepherd, & Hukins, 2008) while that of the respective BC/PMMA composite aerogel was 122 MPa cm^3^ g^−1^.

Compared to pure BC aerogels, the highest gain in specific modulus was achieved for PMMA80 (4.8-fold) and PMMA120 (5.5-fold). For BC/PLA aerogels the specific modulus fell below that of the pure BC aerogel (increase in density without a reinforcing effect) and exceeded it only after the interpenetrating network had been fully developed within the BC network (2.8-fold for PLA120).

Reinforcement with cellulose acetate was the only variant that afforded products with *E*_ρ_ values increasing nearly linearly, starting already from the lowest loading (CA10). This indicates that at contents of the secondary constituent, comparable to the mass of the low density BC structure itself, CA has the highest supporting function among the applied polymers, promoting adhesion of BC fibril junctions already at low concentrations (Table 1). At the highest loading level the obtained composite aerogels (CA120) featured a 3.2-fold multiplication of specific modulus compared to pure BC.

Compared to silica aerogels which are already commercialized for high-performance thermal insulation (Nanogel^®^, Spaceloft^®^), the E-module of BC/CA and BC/PMMA composite aerogels is not only significantly higher at comparable density, but also responds much stronger to changes in density (Fig. 6D). The specific moduli of low density silica aerogels are clearly outmatched by the respective BC/CA composite aerogels. While *E*_ρ_ of BC/CA composite aerogels equaled 50 MPa cm^3^ g^−1^ at a bulk density of 84.2 mg cm^−3^, that of a comparable silica aerogel was about 4 MPa cm^3^ g^−1^ (Alaoui, Woignier, Scherer, & Phalippou, 2008).

The applicability of the investigated reinforcement strategy for BC aerogels was also tested for aerogels obtained by coagulation of cellulose from solution state, basically following a method described elsewhere (Hoepfner, Ratke, & Milow, 2008). In brief, a small amount of cotton linters was dissolved in calcium thiocyanate octahydrate at 140 °C affording a 1 wt% cellulose solution. Coagulation of cellulose (‘regeneration’) was then accomplished by addition of ethanol. Following exhaustive salt leeching with water and solvent exchange to acetone, the reinforcing process was carried out as described for BC samples CA40 and CA80. Accordingly, the samples were labeled rCA40 and rCA80. SEM analyzes revealed that, apart from the higher density of the cellulose mesh, the resulting composites featured open porous morphologies similar to their BC counterparts. Compared to the BC/CA aerogels, the specific moduli *E*_ρ_ of the CA-reinforced aerogels from regenerated cellulose were found to be significantly higher. While *E*_ρ_ was twice as high for rCA40, the next loading level (rCA80) afforded materials whose specific moduli exceeded that of the respective CA80 samples by a factor of three (Table 1).

### 3.4 Pore characteristics

As previously discussed, bacterial cellulose is a material of hierarchical architecture comprising micro-, meso- and macropores. According to thermoporosimetry measurements (data not shown) the peak size distribution peaks in the range of small macropores (ca. 80–100 nm). Nitrogen sorption experiments at 77 K confirmed that the formation of a secondary polymer network affords materials of lower specific surface area (SSA), compared to the specific surface area of BC aerogels (77 m^2^ g^−1^, see Table 2; calculated from the desorption branch of the isotherm). As this is primarily due the higher density of the composite aerogels, the surface area was related to sample volume (surface-area-to-volume ratio; SA_V_), rather than to mass. It was evident that, with the exception of PMMA20 and PMMA40, all BC composite aerogels featured significantly higher SA_V_ values (1.1–6.0 × 10^6^ m^2^ m^−3^) than the BC aerogels (0.7 × 10^6^ m^2^ m^−3^). For BC/PLA, BC/CA, and BC/PMMA aerogels the highest SA_V_ values were obtained for the 120 mg mL^−1^ loading concentration level. In general, the far-reaching maintenance or even enhancement of the surface-area-to-volume ratio confirms the preservation of the aerogel's open-porous morphology throughout the anti-solvent precipitation and drying procedures.

## 4 Conclusions

Loading of PLA, CA, and PMMA from solutions in THF or acetone onto bacterial cellulose, followed by anti-solvent precipitation of the respective polymer inside the highly porous BC organogels and subsequent scCO_2_ drying have been demonstrated to afford BC composite aerogels of significantly enhanced mechanical resistance towards compressive stress at far-reaching preservation of the open-porous BC morphology.

The use of BC (or aerogels from regenerated cellulose) as temporary scaffold for the creation of porous PMMA aerogels, with morphologies resembling the guiding host network, as demonstrated in this work, is an interesting approach which will be further followed in future studies.

## Figures and Tables

**Table 1:** Density and mechanical properties (*n* = 3) under uniaxial compression (rCA samples for comparison: Aerogels obtained by cellulose coagulation from Ca(SCN)_2_·8H_2_O solution, CA loading from acetone and subsequent scCO_2_ anti-solvent precipitation and drying).

	Density (*n* = 4)	*E* (mg cm^−3^)	*E*_ρ_ (MPa cm^3^ g^−1^)	*R*_p0.2_ (kPa)
BC (ethanol)	7.8 ± 0.5^a^	0.15 ± 0.6^a^	19	7.1 ± 0.6^a^
BC (THF)	9.6 ± 0.8	0.20 ± 0.01	21	8.6 ± 0.3
BC (acetone)	9.4 ± 0.5	0.24 ± 0.01^a^	25	13.0 ± 3.0^a^

PCL10	22.2 ± 0.4	0.20 ± 0.06	9	13.7 ± 1.3
PCL20	33.4 ± 1.6	0.44 ± 0.20	13	20.5 ± 8.0

PLA10	15.9 ± 2.1	0.33 ± 0.17	21	13.1 ± 2.1
PLA20	25.1 ± 4.0	0.13 ± 0.01	5	15.9 ± 2.8
PLA40	44.2 ± 2.6	0.21 ± 0.03	5	14.5 ± 2.3
PLA80	106.4 ± 9.8	2.51 ± 0.62	24	279.4 ± 145.5
PLA120	162.0 ± 12.1	9.27 ± 0.33	57	687.4 ± 187.2

CA10	20.1 ± 1.1	0.78 ± 0.16	39	28.2 ± 1.6
CA20	28.8 ± 1.5	1.18 ± 0.26	41	46.6 ± 4.1
CA40	46.6 ± 1.0	2.14 ± 0.44	46	88.1 ± 16.1
CA80	84.2 ± 2.0	4.17 ± 0.27	50	181.6 ± 11.6
CA120	145.3 ± 12.3	11.77 ± 1.67	81	417.8 ± 22.9

PMMA10	18.6 ± 0.9	0.45 ± 0.09	24	23.1 ± 3.8
PMMA20	28.0 ± 0.6	0.67 ± 0.21	24	51.7 ± 10.1
PMMA40	53.2 ± 1.8	1.90 ± 0.65	36	108.1 ± 36.0
PMMA80	100.2 ± 4.9	12.19 ± 1.83	122	485.7 ± 95.1
PMMA120	169.5 ± 0.6	23.58 ± 4.87	139	1513.1 ± 245.9

rCA0	21.0 ± 0.3	0.8 ± 0.1^b^	37	38.6 ± 4.6^b^
rCA40	64.3 ± 3.9	5.8 ± 1.0^c^	90	273.1 ± 57.4^c^
rCA80	85.9 ± 5.1	13.1 ± 2.2^c^	152	403.0 ± 57.3^c^

Sample size: a: *n* = 5, b: *n* = 6, c: *n* = 4.

**Table 2:** Influence of aerogel composition on specific surface area and surface-area-to-volume ratio.

	SSA (m^2^ g^−1^)					SA_V_ (10^6^ m^2^ m^−3^)				
	0	20	40	80	120	0	20	40	80	120
BC	77					0.74				
PCL		54					1.81			
PLA		49	38	25	37		1.23	1.62	2.63	6.00
CA		70	51	25	18		2.03	2.35	2.09	2.69
PMMA		19	10	22	16		0.35	0.27	1.14	1.60
