# Supplementary material for: Reinforcement of bacterial cellulose aerogels with biocompatible polymers
Source: Carbohydr Polym. 2014 Oct 13;111(100):505–13. doi: 10.1016/j.carbpol.2014.04.029 (PMC4118683; doi:10.1016/j.carbpol.2014.04.029)
Supplement: Supplementary file 1 [file mmc1.docx]

SUPPORTING INFORMATION

Reinforcement of bacterial cellulose aerogels with biocompatible polymers

***Pircher, N.^a^, Veigel, S.^b^, Aigner, N.^a,c^, Nedelec, J.-M.^d^, Rosenau, T.^a^, Liebner, F.^a,^****

^a^ University of Natural Resources and Life Sciences Vienna, Division of Chemistry of Renewables, Konrad-Lorenz-Straße 24, A-3430 Tulln, Vienna, Austria

^b^ University of Natural Resources and Life Sciences Vienna, Department of Wood Science, Konrad-Lorenz-Straße 24, A-3430 Tulln, Vienna, Austria

^c^ Swiss Federal Institute of Technology Zurich, Institute for Building Materials, Schafmattstraße 6, 8093 Zurich, Switzerland (current affiliation)

^d^ École Nationale Supérieure de Chimie de Clermont-Ferrand, Laboratoire des Matériaux

Inorganiques, 24 Avenue des Landais, 63177 Aubière, France

* Corresponding author: E-mail: falk.liebner@boku.ac.at, telephone: +43-1-47654-6452


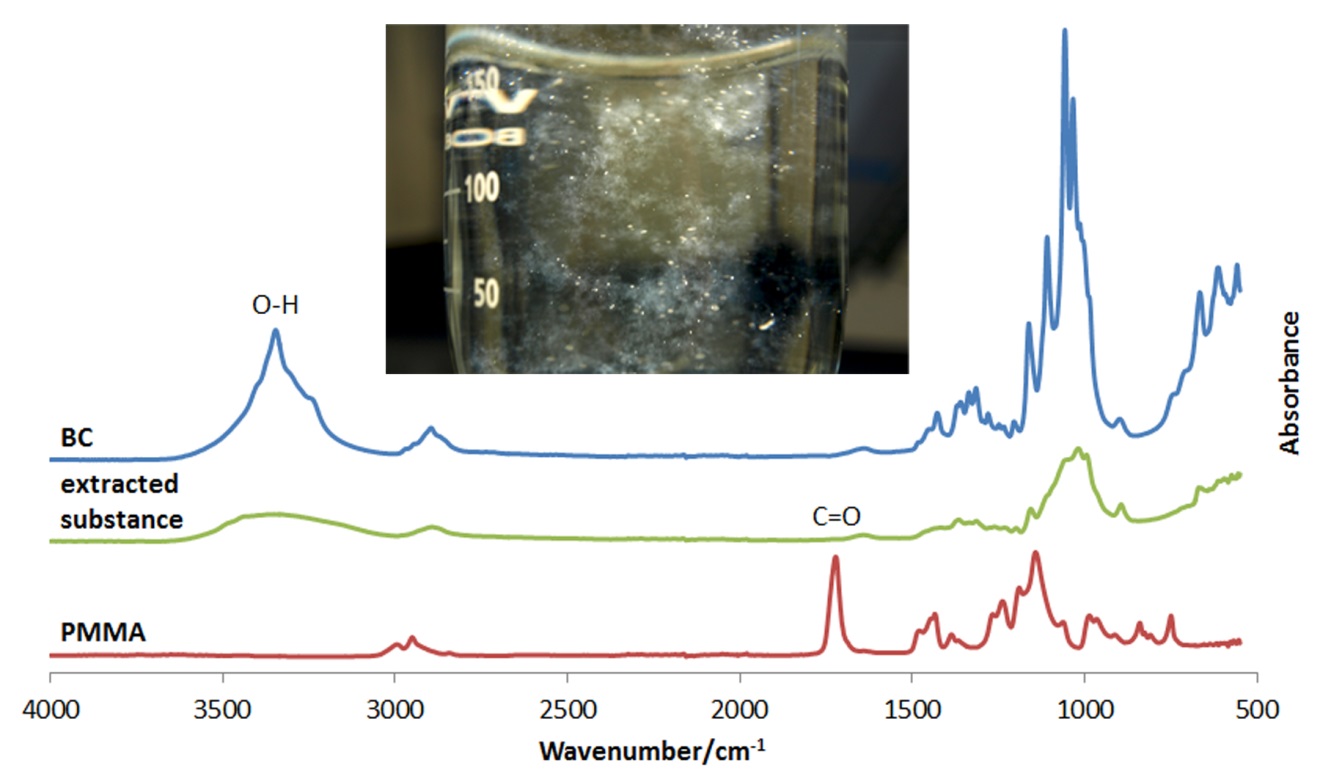


Figure S1: ATR-IR spectra of pure bacterial cellulose (BC) and PMMA. ATR-IR analysis of a substance extracted from BC/PMMA organogels during 5 days immersion in EMIM acetate at 50°C and subsequent antisolvent precipitation (inserted image) and washing using deionized water, revealed exclusive extraction of cellulose (green spectrum).


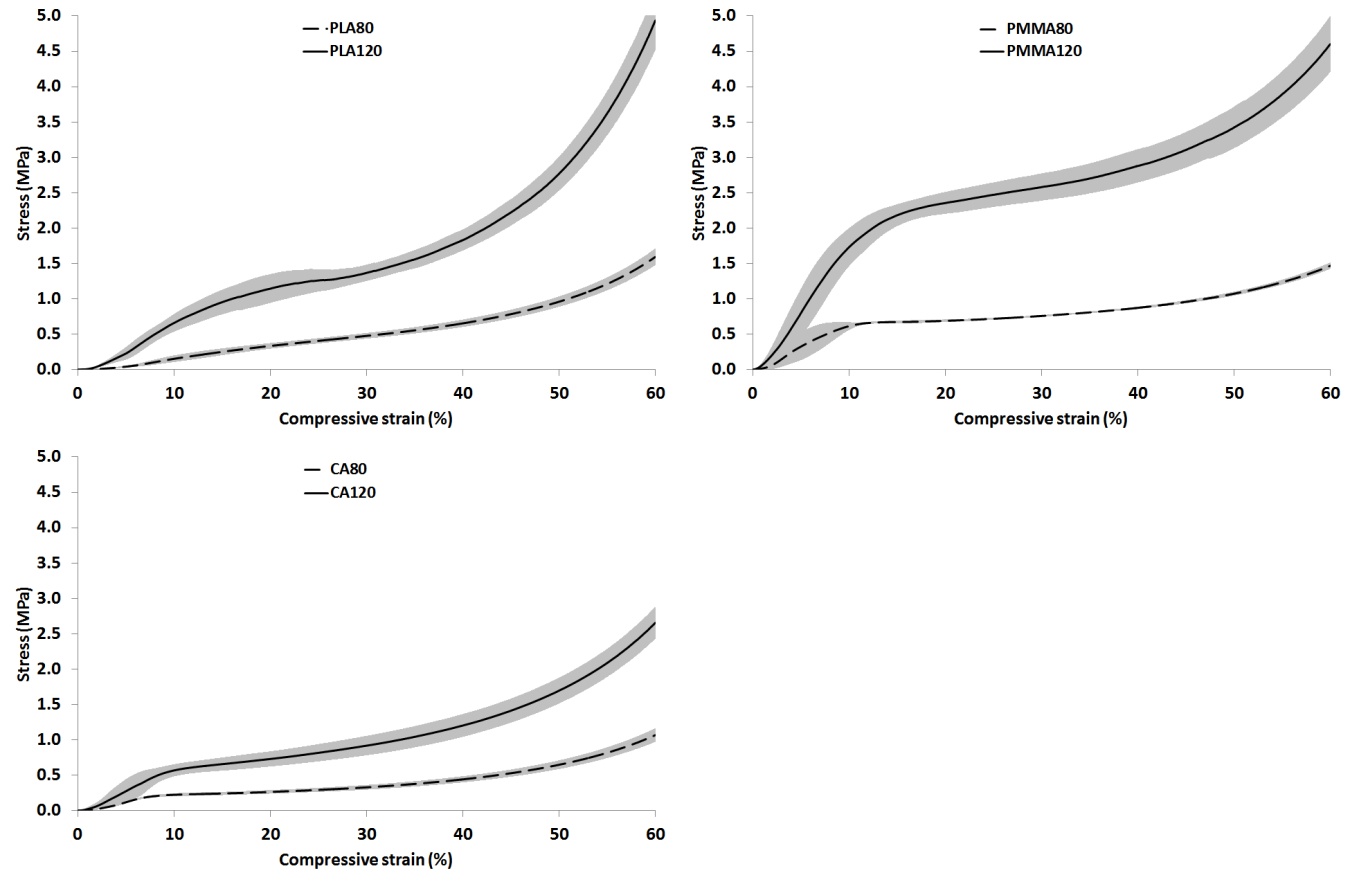


Figure S2: Full mechanical response profiles towards compressive stress for those BC aerogels that were reinforced with high amounts of PLA (A), PMMA (B) and CA (C). Grey areas indicate standard deviations.
